# Neuroprotective Effects of Human Umbilical Cord-Derived Mesenchymal Stem Cells From Different Donors on Spinal Cord Injury in Mice

**DOI:** 10.3389/fncel.2021.768711

**Published:** 2022-01-11

**Authors:** Xu Zhu, Zhen Wang, Yi Eve Sun, Yuchen Liu, Zhourui Wu, Bei Ma, Liming Cheng

**Affiliations:** ^1^Division of Spine, Department of Orthopedics, Tongji Hospital, Tongji University School of Medicine, Tongji University, Shanghai, China; ^2^Key Laboratory of Spine and Spinal Cord Injury Repair and Regeneration, Tongji University, Ministry of Education, Shanghai, China; ^3^Stem Cell Translational Research Center, Tongji Hospital, Tongji University, School of Medicine, Shanghai, China

**Keywords:** human umbilical cord-derived mesenchymal stem cells (hUCMSCs), spinal cord injury, heterogeneities, *Zbtb16*, *Per3*, *Hif3a*

## Abstract

Spinal cord injury (SCI) is caused by an external force, leading to severe dysfunction of the limbs below the injured segment. The inflammatory response plays a vital role in the prognosis of SCI. Human umbilical cord mesenchymal stem cell (hUCMSC) transplantation can promote repair of SCI by reducing the inflammatory response. We previously showed that hUCMSCs from 32 donors had different inhibitory abilities on BV2 cell proliferation. In this study, three experimental groups were established, and the mice were injected with different lines of hUCMSCs. Hind limb motor function, hematoxylin-eosin (H&E) staining, immunohistochemistry, Western blot (WB), qualitative real-time polymerase chain reaction (qRT-PCR), and RNA sequencing and correlation analysis were used to investigate the effects of hUCMSC transplantation on SCI mice and the underlying mechanisms. The results showed that the therapeutic effects of the three hUCMSC lines were positively correlated with their inhibitory abilities of BV2 cell proliferation rates *in vitro*. The MSC_A line had a better therapeutic effect on improving the hind limb motor function and greater effect on reducing the expression of glial fibrillary acidic protein (Gfap) and ionized calcium binding adaptor molecule 1 (Iba1) and increasing the expression of neuronal nuclei (NeuN). Differentially expressed genes including *Zbtb16*, *Per3*, and *Hif3a* were probably the key genes involved in the protective mechanism by MSC_A after nerve injury. qRT-PCR results further verified that *Zbtb16, Per3*, and *Hif3a* expressions reduced by SCI could be reversed by MSC_A application. These results suggest that the effect of hUCMSCs transplantation on acute SCI depends on their inhibitory abilities to inflammation reaction after nerve injury, which may help to shape future use of hUCMSCs combined with improving the effectiveness of clinical transformation.

## Introduction

Spinal cord injury (SCI) is one of the most devastating central nervous system diseases and can cause severe nerve dysfunction and, at times, paralysis (Rogers and Todd, 2016). At present, there is no effective treatment for SCI. SCIs are heavily burdensome to the families of patients and society on account of the high morbidity, high disability rate, and high cost (Singh et al., 2014; Reinhardt et al., 2017; Badhiwala et al., 2019; Quadri et al., 2020). The pathology of SCI is divided into two stages, namely, primary injury that refers to the direct damage by an external force, and the following secondary injury that lasts for a period caused by ischemia and hypoxia of the damaged segment, inflammation, local edema, and metabolic disorder (Oyinbo, 2011). The inflammatory response has been considered an important pathological process that is closely related to the prognosis of SCI. Under physiological conditions, immunocytes cannot pass the blood-spinal cord barrier; microglia, regarded as the major innate immune cells of the spinal cord (Anwar et al., 2016), are quickly activated upon damage to the spinal cord. The injury of the blood-spinal cord barrier after SCI induces neutrophils, monocytes, and macrophages to enter the injured spinal cord (Beck et al., 2010; Aubé et al., 2014; Gensel and Zhang, 2015; Jin et al., 2021). Many inflammatory factors are released, which further activate the microglia in damaged spinal cord tissues, triggering a severe local immune response. Lots of neurons are destroyed after SCI, and this is followed by the formation of subsequent glial scar, which finally leads to serious secondary injury (Zhang et al., 2016). Therefore, controlling the activation of microglia and the resultant production of inflammatory factors may contribute to ameliorating the microenvironment of regeneration in SCI (Mizuno, 2011; Zhang et al., 2013).

Stem cell therapy has been considered as a potential method for SCI treatment (Assinck et al., 2017). As the origin of human umbilical cord mesenchymal stem cell (hUCMSCs), human umbilical cords (hUCs) are discarded tissues that are found in abundant supply. hUCMSCs can be obtained using a non-invasive method, and they have low immunogenicity, high immune tolerance, and stemness properties (Ding et al., 2015). hUCMSCs have been regarded as having the most potential for the treatment of injury to the central nervous system due to their features (Cao and Feng, 2009; Alessandrini et al., 2019). Many cell and animal experiments proved that hUCMSCs had a stable immunomodulatory function, and transplantation of hUCMSCs can promote the recovery of SCI by inhibiting the immune-inflammatory response (Aggarwal and Pittenger, 2005; Riordan et al., 2019; Yang et al., 2020). It has been reported that hUCMSCs from different tissues have different qualities, as indicated by dramatic differences in their immunosuppressive effects (Huang et al., 2013). Therefore, the therapeutic effects of hUCMSCs derived from different donors on SCI might be different while the underlying mechanisms remain unclear.

In our previous study, we found that hUCMSCs from different donors (e.g., mothers) showed different inhibitory abilities for BV2 cell proliferation (Zhang et al., 2021). We selected three cell lines that had strong, general, and weak inhibition to BV2 cell proliferation, labeled as line A of human umbilical cord-derived mesenchymal stem cell (MSC_A), line B of human umbilical cord-derived mesenchymal stem cell (MSC_B), and line C of human umbilical cord-derived mesenchymal stem cell (MSC_C), and hypothesized that the hUCMSC line that had a strong inhibition to BV2 cell proliferation would have a greater therapeutic effect on SCI than the other lines through inhibition of the microglia-mediated inflammatory response. After a model of spinal cord clamp injury was established in mice, MSC_A, MSC_B, and MSC_C cells were transplanted intravenously at different time windows (i.e., dpo 1, dpo 3, and wpo 2) to determine which time point and which cell line was more beneficial for the recovery of SCI. We further detected the level of SCI-related proteins and mRNA by Western blot and q-PCR when hUCMSCs were transplanted at dpo 3. Finally, we searched for the key genes of hUCMSC treatment for SCI through mRNA-sequencing and verified the results with q-PCR.

## Materials and Methods

### Establishment of Mice Model of Spinal Cord Injury

A total of 156 adult female C57 mice, weighing 20–25 g, SPF grade, were granted to us by Tongji Hospital Laboratory Animal Center (20070010). The local legislation for the ethics of experiments on animals and guidelines for their care and the use of laboratory animals were followed in all animal procedures. All mice were housed in separated cages with free access to food and water; the room had the right temperature and humidity, and there was natural illumination before and after the surgery. All mice were given a week to adapt to the laboratory environment before the experiment.

Mice were positioned on a thermistor-controlled heating pad in a prone position after being anesthetized with pentobarbital (50 mg/kg, i.p.). The skin above the vertebral column was shaved and cleaned with alcohol. A skin incision was made on the midline of the back that exposed the paravertebral muscles. These muscles were separated, and the spinous process of T9 was exposed with a pair of fine ophthalmic scissors. The spinal cord was exposed with the dura remaining intact *via* a T9 laminectomy. The operating rod was connected with special tweezers (Dumont#5 0.01 × 0.05) and fixed on a stereotaxic apparatus. It was carefully placed on the front end of the forceps on both sides of the exposed T9 spinal cord, down about 0.5 mm until it touched the bottom vertebral bone. Then, the forceps were completely clamped. The distance between the tip of the forceps and the spinal cord was 0 mm. The clamp was held for 3 s, and then the wound was sutured. The following symbols are indicators of a successful SCI model: a complete clamp trace occurs, and paralysis of both lower limbs occurs after awakening. Unsuccessful models were excluded from the experiment. In the early postoperative period, the mouse bladders were manually voided two times a day until the mice were able to regain the normal bladder function.

The mice were randomly assigned into the following groups: the sham group, the SCI group, and the hUCMSC treatment group (including MSC_A, MSC_B, and MSC_C groups). Mice in the sham group only underwent laminectomy and exposure of the spinal cord, but there was no injury; mice in the SCI group had spinal cord injuries as described above and 100 μl saline injection through a tail vein; the hUCMSC treatment groups were then divided into nine groups; the three lines (i.e., MSC_ A, MSC_B, and MSC_C) were transplanted *via* the tail vein at a dose of 1 × 10^6^/100 μl at 1 day post operation (dpo 1), 3 days post operation (dpo 3), and 2 weeks post operation (wpo 2).

### Western Blot

The mice were sacrificed after being anesthetized with 1% pentobarbital (40 mg/kg, i.p.) to obtain a 0.5 cm section of the spinal cord containing the compression injury site at 1 week and 1 month after the hUCMSC treatment. Then, the spinal cord tissues were rinsed with phosphate-buffered saline (PBS) under sterile conditions and homogenized in the ice-cold lysis buffer (RIPA lysis buffer kit, phenylmethylsulfonyl fluoride 0.1 mM). Tissue lysates were centrifuged at 12,000 rpm for 30 min, and the supernatant was collected. Protein concentrations were determined using a bicinchoninic acid (BCA) protein assay kit according to the instructions of the manufacturer. Samples were diluted in sample buffer and boiled for 20 min. Proteins (20 ng) from each sample were loaded onto 12% polyacrylamide gels and separated using electrophoresis. Separated proteins were then transferred to polyvinylidene difluoride membranes. After blocking with 5% non-fat dry milk for 2 h at 4°C, membranes were probed with the primary antibodies overnight, i.e., mouse anti-neuronal nuclei (NeuN) (1:1,000), rabbit anti-glial fibrillary acidic protein (Gfap) (1:1,000), rabbit anti-ionized calcium binding adaptor molecule 1 (Iba1) (1:1,000), and rabbit anti-β-actin (1:1,000), followed by three times of washing with PBS for 10 min each and then incubation with the proper secondary antibody (horseradish peroxidase-conjugated goat anti-rabbit/mouse secondary antibody) for 2 h at room temperature. Protein bands were visualized with enhanced chemiluminescence (ECL) after washing with buffer three times for 10 min each again. Then, images were acquired and analyzed with the FR-200 system. The relative protein expression was normalized to β-actin.

### Hematoxylin-Eosin Staining

After being anesthetized, the mice were fixed in a supine position, the chest was cut open, and the heart was exposed under direct vision. A syringe was inserted into the left ventricle, and the auricula dextra was exposed. The mice were rapidly perfused with 20 ml PBS, and 20 ml 4% paraformaldehyde (PFA) was slowly infused subsequently until the limbs and tail hardened. Spinal cords containing the epicenter of the lesion were dissected out and kept in 4% PFA overnight at 4°C. Then, after dehydration with 10, 20, and 30% sucrose successively for 3 days, the spinal cords were embedded with optimal cutting temperature compound, and serial transverse frozen sections with a thickness of 10 μm were obtained. The frozen sections were dried in air for 1 h and then washed briefly in PBS three times for 5 min, immersed in hematoxylin solution for 1 min, and finally washed with water. The sections were then treated with 1% hydrochloric acid for 10 s, washed, and then submerged in 0.5% eosin for 2 min. The sections were washed again and dehydrated in gradient alcohol, made transparent in dimethyl benzene, and sealed with neutral resin. Finally, they were scanned with a microscope.

### Immunofluorescence

The spinal cord frozen sections were dried in air for 1 h and then washed in PBS three times for 5 min each. Sections were immersed in 0.04% Triton for 20 min. Next, the sections were incubated with a blocking reagent for 1 h at room temperature and further incubated with primary antibodies including anti-NeuN (1:500), anti-Gfap, and anti-Iba1 (1:500) antibodies at 4°C overnight. The next day, samples were immersed with goat-rabbit/mouse IgG secondary antibodies (1:1,000) for 2 h after washing with phosphate-buffered saline with Tween 20 (PBST). Then, the sections were counterstained with 4′,6-diamidino-2-phenylindole (1:1,000) to identify the cell nuclei. After being washed and sealed, the sections were imaged with a laser scanning confocal microscope.

### Behavioral Test of Locomotor Function

The Basso Mouse Scale (BMS) is a sensitive, reliable, and valid test for assessing the locomotor recovery of the mouse following SCI (Basso et al., 2006). It categorizes combinations of mice hindlimb movements including ankle movements, weight support, plantar stepping, trunk position and stability, tail position, and so on, representing sequential recovery stages that mice attain after SCI. A locomotor rating scale of 0 (i.e., complete paralysis) to 9 (i.e., normal locomotion) was used. BMS scores were recorded at 1, 2, 3, 4, 5, and 6 wpo. Mice were given 5 min to adapt to the environment before each test, and each assessment lasted more than 5 min.

### Culture of Human Umbilical Cord Mesenchymal Stem Cells and Transplantation

hUCMSCs were prepared by the Stem Cell Engineering Research Center of the Tongji Hospital, Tongji University, under ethical approval, including informed written consent from donors. The protocol for the isolation and expansion of hUCMSCs was previously reported. Briefly, the cord blood was removed after fresh UCs were rinsed three times in Hank’s balanced salt solution (HBSS), disinfected in 75% alcohol for 10 s, and then washed again with HBSS. The cords were cut into two 1 mm pieces and floated in Dulbecco’s modified Eagle’s medium/F12 medium containing 10% fetal bovine serum, and 1% Penicillin/Streptomycin (P/S) (v/v) after filtering and incubating at 37°C in humidified air consisting of 5% CO_2_. Non-adherent cells were removed by washing with PBS. The medium was replaced every 3 days after the initial plating until the monolayer of hUCMSCs colonies appeared as fibroblast-like cells. When most cells were seeded to confluence, the cells were trypsinized, washed, resuspended, and passaged into a new plate for further expansion, and the medium was changed every 3 days. Cells of the fifth passage of each hUCMSC line that was harvested were used for further experiments. The hUCMSCs were centrifuged after counting and diluted in PBS at a dose of 1 × 10^6^/100 μl PBS. In the hUCMSC treatment groups, at 1 day, 3 days, and 2 weeks after SCI, 1 × 10^6^ hUCMSCs were injected slowly through the tail vein of the mouse for 2 min, and the injection point was pressed for 1 min before putting the mouse back into the cage.

### Total RNA Extraction

The tissues were crushed and placed for 5 min in TRIzol. Then, the mixture was centrifuged at 12,000 × *g* for 5 min at 4°C. The supernatant was transferred to a new Eppendorf tube with a moderate dose of chloroform. The mixture was shaken vigorously for 15 s and then centrifuged at 10,000 × *g* for 10 min at 4°C. Then, the RNA retaining the upper aqueous phase was transferred to a new tube with an equal volume of isopropanol supernatant and centrifuged at 10,000 × *g* for 20 min at 4°C. After discarding the supernatant, the RNA particles were washed twice with 75% ethanol; the residual ethanol was removed after being centrifuged at 13,600 rpm for 3 min at 4°C, and then, the particles were dried in the biosafety cabinet for 10 min. The RNA was dissolved by adding 30 μl diethyl pyrocarbonate-treated water. Finally, the total RNA was qualified and quantified.

### Quantitative Real-Time Polymerase Chain Reaction

Levels of mRNA were analyzed using the qualitative real-time polymerase chain reaction (qRT-PCR). The method has been described in previous studies (Zhu et al., 2021). Briefly, RNA was determined after isolated as describe above. The qualified mRNA was reversely transcribed into the complementary deoxyribonucleic acid (cDNA) with the reverse transcription kit (PrimeScript RT reagent Kit, TaKaRa, Japan, Code No. RR047A), followed by qRT-PCR using TB Green Advantage qPCR premixes (TaKaRa, Japan, Code No. RR820A). Finally, Gapdh was used for housekeeping gene control; a relevant level of the target gene was analyzed using the 2^–ΔΔ*CT*^ method. The primers were as follows:

**Table:** 

The primers	The primer pairs
Il7r-fp	GCGGACGATCACTCCTTCTG
Il7r-rp	AGCCCCACATATTTGAAATTCCA
Per3-fp	AAAAGCACCACGGATACTGGC
Per3-rp	GGGAGGCTGTAGCTTGTCA
Cd200r4-fp	GTCTATGACCTCCAAGTGCTGG
Cd200r4-rp	GTGCCATTGCTGTGTGACTCAC
Nrk-fp	GACCTGGGAGTTGGAGGGA
Nrk-rp	CATAAGTACCAAGACCAATGGCT
Zbtb16-fp	CTGGGACTTTGTGCGATGTG
Zbtb16-rp	CGGTGGAAGAGGATCTCAAACA
Hif3a-fp	GAAGTTCACATACTGCGACGA
Hif3a-rp	GTCCAAAGCGTGGATGTATTCAT
Gfap-fp	CCCTGGCTCGTGTGGATTT
Gfap-rp	GACCGATACCACTCCTCTGTC
Iba1-fp	TCTGCCGTCCAAACTTGAAGCC
Iba1-rp	CTCTTCAGCTCTAGGTGGGTCT
NeuN-fp	GGCTGAGCATATCTGTAAGCTGC
NeuN-rp	GGCTGAGCATATCTGTAAGCTGC
Gapdh-fp	CATCACTGCCACCCAGAAGACTG
Gapdh-rp	ATGCCAGTGAGCTTCCCGTTCAG

### MRNA Library Construction

The mRNA was purified with oligo(dT)-attached magnetic beads and then crushed into small pieces with a fragment buffer at the appropriate temperature. Using random hexamer-primed reverse transcription, the first-strand cDNA was generated, followed by second-strand cDNA synthesis. Subsequently, a tail mixture and RNA index adaptor were added for end-repair after incubation. The cDNA fragments were amplified by PCR after being obtained with the previous step. Ampure XP Beads and EB solution were used to purify and dissolve the products. An Agilent Technologies 2100 bioanalyzer was used to validate the product for quality control. The final library was obtained after heating, denaturing, and circularizing the double-stranded PCR products with the splint oligo sequence. The final library was built by formatting the single-strand circular DNA and then amplifying with phi29 to make DNA nanoballs (DNBs), which had more than 300 copies of one molecule. DNBs were loaded into the patterned nanoarray, and single-end 50 base reads were generated on the BGIseq500 platform (BGI-Shenzhen, China).

### Sequencing Analysis

The sequencing data were filtered with SOAPnuke by removing reads once meeting the following three conditions, namely, (1) reads containing sequencing adapter; (2) reads whose low-quality base ratio is more than 20%; and (3) reads whose unknown base ratio is more than 5%, and afterward clean reads were obtained and stored in the FASTQ format (Li et al., 2008). HISAT2 was used for mapping the clean reads to the reference genome. Bowtie2 was applied to align the clean reads to the reference coding gene set; RNA-sequencing by expectation maximization calculated the expression level of the gene (Li and Dewey, 2011). The heatmap was drawn by pheatmap according to the gene expression in different samples. Essentially, the differential expression analysis was performed using the DESeq2 with *Q* value ≤ 0.05 (Love et al., 2014). The Gene Ontology and Kyoto Encyclopedia of Genes and Genomes (KEGG) enrichment analysis of annotated different expressed gene was performed using Phyper based on the hypergeometric test to take insight to the change of phenotype. The significant levels of terms and pathways were corrected by *Q* value with a rigorous threshold (*Q* value ≤ 0.05) by Bonferroni.

### Statistical Analysis

For comparisons of three or more groups, the one-way ANOVA with *post hoc* Tukey’s test was utilized. The data were expressed as the mean + SEM. A significant difference was defined as ^∗^*p* < 0.05 and ^∗∗^*p* < 0.01.

## Results

### Culture, Identification, and Selection of Human Umbilical Cord Mesenchymal Stem Cells

**FIGURE 1:**
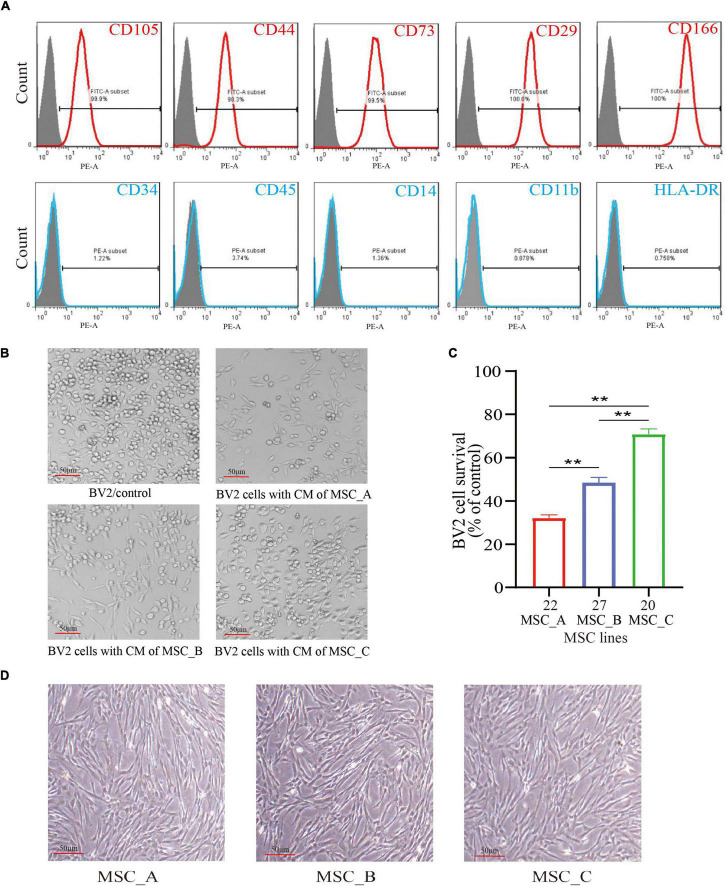
Culture, identification, and selection of human umbilical cord mesenchymal stem cells (hUCMSCs). **(A)** Flow cytometry analysis of surface markers in hUCMSCs. **(B)** The BV2 cells were cultured with the culture medium (CM) of MSC_A, MSC_B, and MSC_C for 48h. **(C)** Different lines of hUCMSCs had different inhibitory abilities on the proliferation rate of BV2 cells. **(D)** The three lines of hUCMSCs attached to the wall and sprawled out, which exhibited a fibroblast-like shape. All data are presented as the mean ± SEM; *n* = 3; *p < 0.05; **p < 0.01.

Flow cytometry was used to examine the surface markers of the hUCMSCs. The results showed that most of the cultured cells were positive for CD105, CD44, CD73, CD29, and CD166 expressions; meanwhile, they were negative for CD34, CD45, CD14, CD11b, and HLA-DR expressions (Figure 1A). The proliferation rate of BV2 cells was detected after co-culturing with the 32 lines of hUCMSCs culture medium. The results showed that different lines of hUCMSCs had different inhibitory abilities to the proliferation of BV2 cells (Zhang et al., 2021). We chose line #22 with a strong inhibitory ability (i.e., MSC_A), line #20 with a weak inhibitory ability (i.e., MSC_C), and line #27 with a moderate inhibitory ability (i.e., MSC_B) for the subsequent experiments (Figures 1B,C). The three lines of hUCMSCs exhibited similar spindle-like and fibroblast-like shapes (Figure 1D).

### Recovery of Motor Function Assessed by Basso Mouse Scale Score After Human Umbilical Cord Mesenchymal Stem Cell Treatment

**FIGURE 2:**
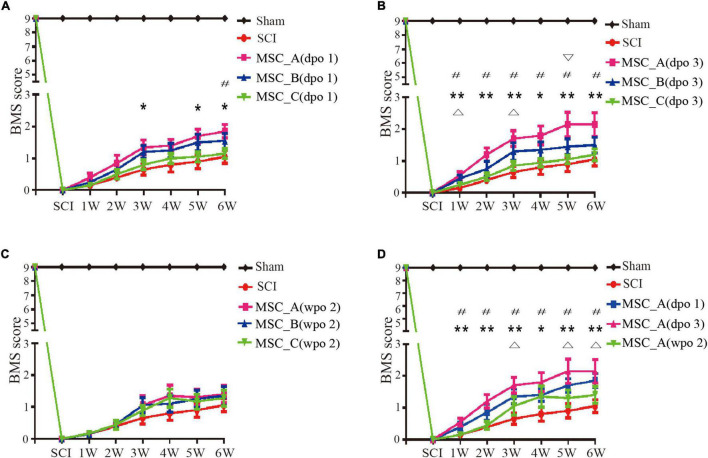
Recovery of the motor function assessed by Basso Mouse Scale (BMS) scores over time. **(A)** The BMS scores were compared with the spinal cord injury (SCI) group when hUCMSC transplantation was performed at dpo 1. *p < 0.05, **p < 0.01 MSC_A group compared with the SCI group; *^#^p* < 0.05 MSC_A group compared with the MSC_C group. **(B)** The BMS scores were compared with the SCI group when hUCMSC transplantation was performed at dpo 3. *p < 0.05 MSC_A group compared with the SCI group; ^#^*p* < 0.05 MSC_B group compared with SCI group; ^#^*p* < 0.05 MSC_A group compared with the MSC_C group; ^∇^ *p* < 0.05 MSC_A group compared with the MSC_B group. **(C)** The BMS scores were compared with the SCI group when hUCMSC transplantation was performed at wpo 2. **(D)** The BMS scores were compared with the SCI group when MSC_A transplantation was performed at different time points including dpo 1, dpo 3, and wpo 2. *p < 0.05, **p < 0.01 MSC_A dpo 3 compared with the SCI group; ^Δ^*p* < 0.05 MSC_A dpo 1 compared with the SCI group; ^#^*p* < 0.05 MSC_A dpo 3 compared with the MSC_A wpo 2.

After clamp injury of the spinal cord, the motor function of hind limbs of the mice was completely lost at dpo 1. Compared with the SCI group, the groups with hUCMSC treatments had a positive therapeutic effect at different time points (dpo 1, dpo 3, and wpo 2). When hUCMSC transplantation was performed at dpo 1, the BMS score was increased significantly compared with that in the SCI group; the increase in the MSC_A group was more obvious than those in the other two MSC groups. Similarly, a higher BMS score was observed for the MSC_A group at wpo 3, wpo 5, and wpo 6 (Figure 2A), whereas there was no significant difference in either the MSC_B or MSC_C group, compared with that in the SCI group. When hUCMSC transplantation was performed at dpo 3, the BMS score was significantly increased compared with that in the SCI group. Similar results were found for the MSC_A group at six recording points from wpo 1 to wpo 6. Moreover, there were statistical differences in the MSC_B group only at wpo 1 and wpo 3. The MSC_A group presented the highest maximum score among the three MSC groups (Figure 2B). When hUCMSC transplantation occurred at wpo 2, there was no statistical difference between the SCI group and the three MSC groups (Figure 2C). When comparing the BMS scores of the MSC_A transplantation at dpo 1, dpo 3, and wpo 2, the increase in BMS scores after MSC_A transplantation at dpo 3 was the highest (Figure 2D).

### The Expressions of Neuronal Nuclei, Glial Fibrillary Acidic Protein, and Ionized Calcium Binding Adaptor Molecule 1 in the Protein Level After Human Umbilical Cord Mesenchymal Stem Cell Treatment at dpo 3

**FIGURE 3:**
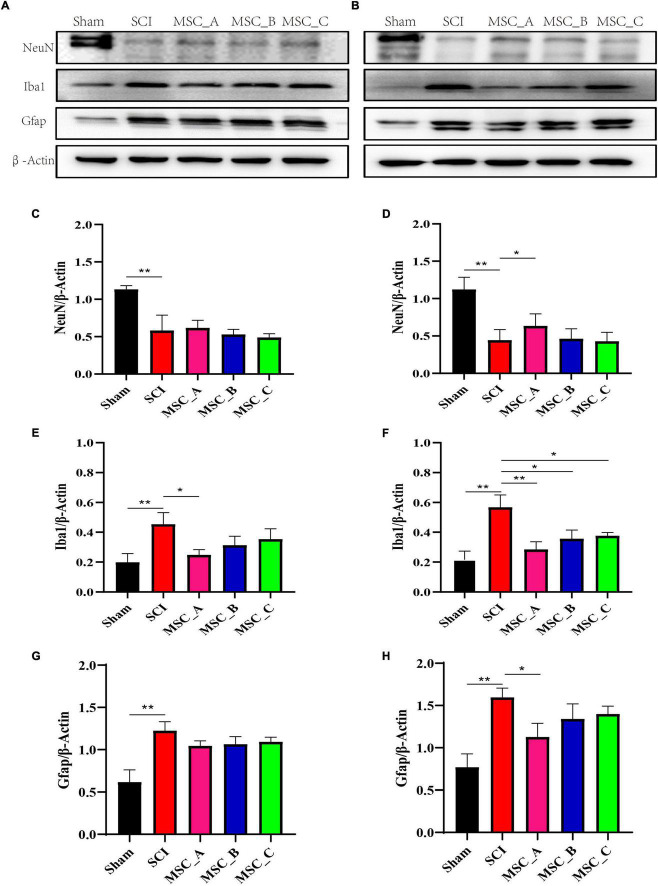
Proteins related to SCI were detected at 1 week and 1 month after hUCMSC treatment at dpo 3. Western blot assay of neuronal nuclei (NeuN), glial fibrillary acidic protein (Gfap), and ionized calcium binding adaptor molecule 1 (Iba1) expressions at 1 week **(A)** and 1 month **(B)** after hUCMSC transplantation. Relative quantitative comparison of interest protein bands that were standardized by β-actin at 1 week **(C,E,G)** and 1 month **(D,F,H)** after hUCMSC transplantation. All data are presented as the mean ± SEM; *n* = 3; *p < 0.05; **p < 0.01.

The injured spinal cord was collected at 1 week and 1 month after transplantation. Western blot was used to detect the NeuN, Gfap, and Iba1 proteins, which are specific proteins of neurons, astrocytes, and microglia. The results in the present study showed that the NeuN protein level was significantly decreased, while the levels of Gfap and Iba1 proteins were significantly increased after SCI (Figures 3A,B), which was consistent with previous results. Compared with that in the SCI group at 1 week after transplantation, the expression of Iba1 protein was significantly decreased only in the MSC_A group but not in the MSC_B or MSC_C group (Figure 3E). There was no statistical difference in the changes in NeuN and Gfap protein levels in the three MSC treatment groups, compared with that in the SCI group (Figures 3C,G). At 1 month after transplantation, compared with that in the SCI group, the content of NeuN protein was enhanced, and the content of Gfap and Iba1 proteins was reduced in all MSC groups. The Iba1 proteins of the three MSC groups were all markedly decreased, among which the change in the MSC_A group was most obvious (Figure 3F). Compared with those in the SCI group, a statistical difference in NeuN and Gfap proteins existed in the MSC_A group (Figures 3D,H) but not in the MSC_B or MSC_C group.

### The Expressions of *NeuN*, *Gfap*, and *Iba1* in the mRNA Level After Human Umbilical Cord Mesenchymal Stem Cell Treatment at dpo 3

**FIGURE 4:**
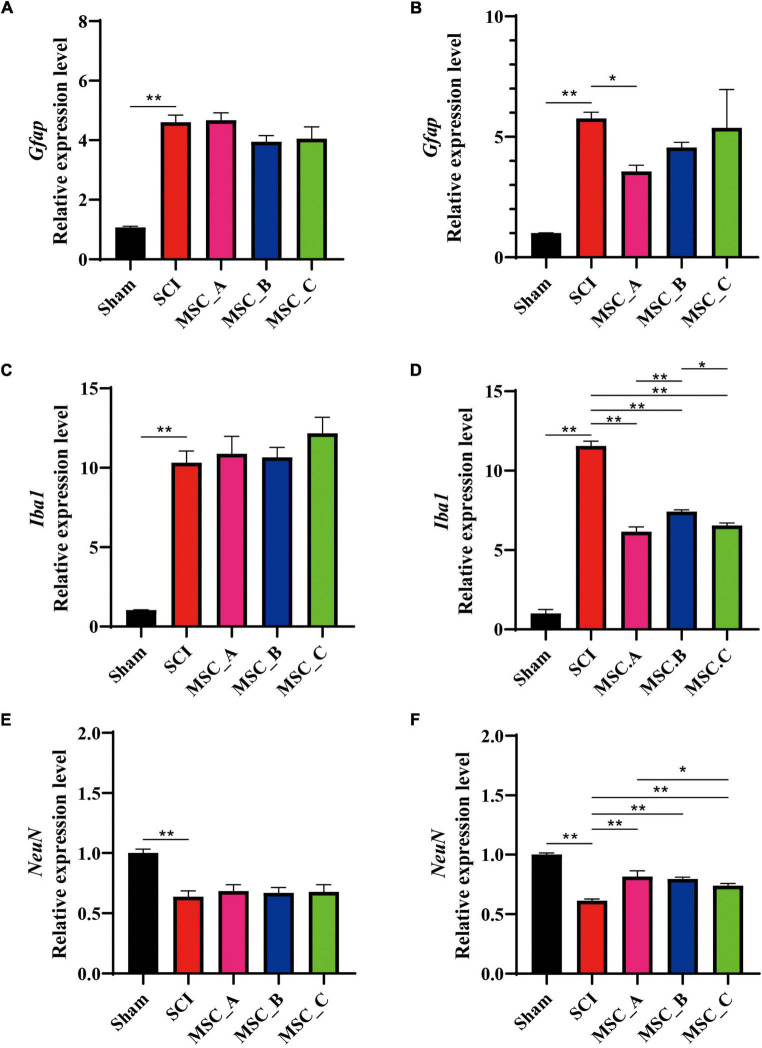
The qualitative polymerase chain reaction (q-PCR) showing the changes in mRNA related to SCI at 1 week and 1 month after hUCMSC treatment at dpo 3. The mRNA expressions of *Gfap* **(A)**, *Iba1* **(C)**, and *NeuN* **(E)** at 1 week after hUCMSC transplantation. The mRNA expressions of *Gfap* **(B)**, *Iba1* **(D)**, and *NeuN* **(F)** at 1 month after hUCMSC transplantation. All data are presented as the mean ± SEM; *n* = 3; *p < 0.05; **p < 0.01.

The q-PCR results showed a similar trend as Western Blot (WB). SCI induced lower mRNA expression of *NeuN* and higher mRNA expression of *Iba1* and *Gfap*, but there was no statistical difference between the SCI group and MSC treatment groups at 1 week after transplantation (Figures 4A,C,E). Compared with that in the SCI group at 1 month after transplantation, the hUCMSC injection decreased the mRNA expression of *Gfap*, a statistical difference only existed in the MSC_A group among the three MSC groups (Figure 4B). The higher expression of *Iba1* in the SCI group was significantly decreased in all three MSC groups. The *NeuN* expressions in all three MSC groups increased obviously when compared with the SCI group. Furthermore, in the MSC_A group, the *Iba1* expression was lower and the *NeuN* expression was higher than those in MSC_B or MSC_C group, respectively (Figures 4D,F).

### Hematoxylin-Eosin and Glial Fibrillary Acidic Protein Immunofluorescence Staining of Spinal Cord After Human Umbilical Cord Mesenchymal Stem Cell Treatment at dpo 3

**FIGURE 5:**
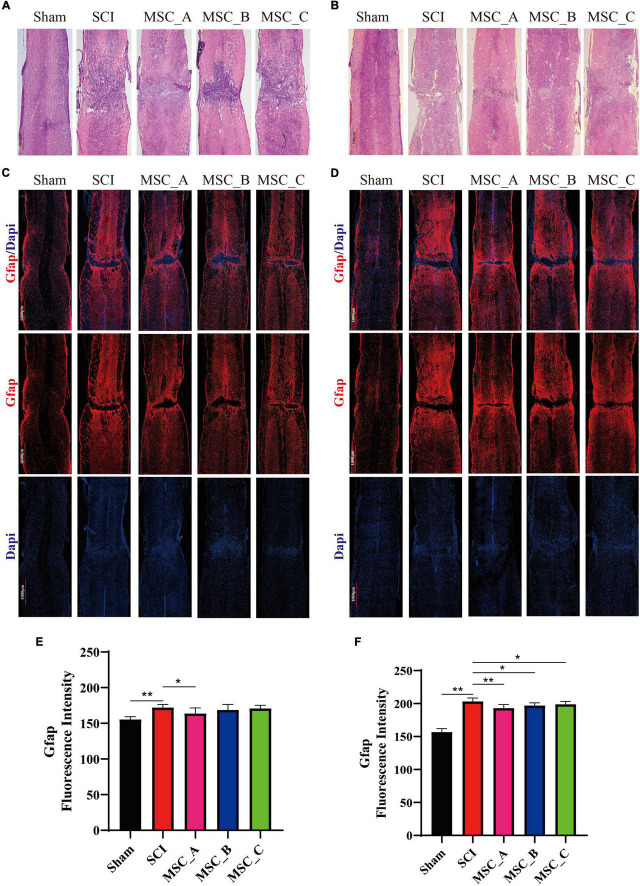
Hematoxylin-eosin and Gfap immunofluorescence staining at 1 week and 1 month after hUCMSC treatment at dpo 3. Gross morphological examination of the spinal cord by H&E staining at 1 week **(A)** and 1 month **(B)** after hUCMSC transplantation. Confocal images of immunostaining confirmed the expression of glial fibrillary acidic protein (Gfap, in red) and nucleus [4′,6-diamidino-2-phenylindole (DAPI), in blue] at 1 week **(C)** and 1 month **(D)** after hUCMSC transplantation. Quantification of the Gfap fluorescence intensity at 1 week **(E)** and 1 month **(F)** after hUCMSC transplantation. All data are presented as the mean ± SEM; *n* = 3; *p < 0.05; **p < 0.01.

Hematoxylin-eosin (H&E) staining showed that the spinal cords of the hUCMSC groups had cavities that were much smaller and tissue morphology integrity that was better than those of the SCI group at 1 week and 1 month post-hUCMSC treatment (Figures 5A,B). Compared with the Sham group, the immunofluorescence image revealed many astrocytes were activated around the injury site in the SCI group, while the activation of astrocytes was significantly reduced after hUCMSC transplantation compared with the SCI group (Figures 5C,D). The fluorescence intensity of Gfap was quantified, and the results showed at 1 week post-hUCMSC transplantation a significant decrease of the Gfap fluorescence intensity was found only in the MSC_A group but not in the MSC_B or MSC_C group (Figure 5E). While at 1 month after hUCMSC transplantation, compared with that in the SCI group, the Gfap fluorescence intensity was reduced significantly in all three MSC groups, among which was most obvious in the MSC_A group (Figure 5F).

### Ionized Calcium Binding Adaptor Molecule 1 and Neuronal Nuclei Immunofluorescence Staining of Spinal Cord After Human Umbilical Cord Mesenchymal Stem Cell Treatment at dpo 3

**FIGURE 6:**
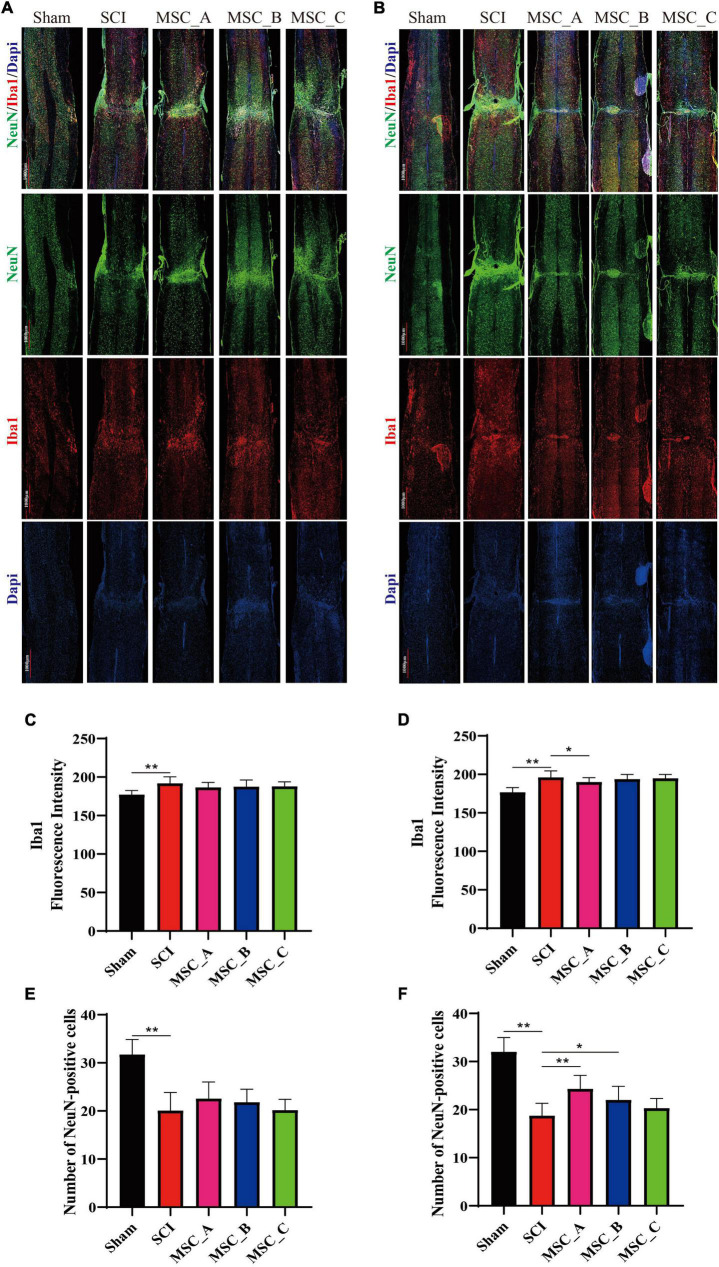
Ionized calcium binding adaptor molecule 1and NeuN immunofluorescence staining at 1 week and 1 month after hUCMSC treatment at dpo 3. Confocal images of immunostaining confirmed the expressions of ionized calcium binding adapter molecule 1 (Iba1, in red), neuron (NeuN, in green), and nucleus (DAPI, in blue) at 1 week **(A)** and 1 month **(B)** after hUCMSC transplantation. Quantification of Iba1 fluorescence intensity at 1 week **(C)** and 1 month **(D)** after hUCMSC transplantation. Quantification of the NeuN-positive cell number at 1 week **(E)** and 1 month **(F)** after hUCMSC transplantation. All data are presented as the mean ± SEM; *n* = 3; *p < 0.05; **p < 0.01.

Compared with those in the Sham group, many microglia were activated and numerous neurons were lost around the injury site in the SCI group. The immunofluorescence image revealed the hUCMSC transplantation significantly reduced the activation of microglia around the injury site compared with that in the SCI group, and there were more NeuN-positive cells around the injury site in all three MSC groups when compared with the SCI group (Figures 6A,B). Hence, we quantified the fluorescence intensity of Iba1 and counted the number of neurons around the injury site. At 1 week post-hUCMSC transplantation, the differences in Iba1 fluorescence intensity and the NeuN-positive number between the SCI group and any one of the three MSC groups were not statistically significant (Figures 6C,E). While at 1 month after hUCMSC transplantation, compared with that in the SCI group, the Iba1 fluorescence intensity was significantly decreased in the MSC_A group but not in the MSC_B or MSC_C group (Figure 6D). When compared with the SCI group, statistical changes in the NeuN-positive number were found in the MSC_A group and MSC_B group but not in the MSC_C group (Figure 6F). The therapeutic effect of MSC_A in SCI mice was obviously better than that in the MSC_B or MSC_C group. Meanwhile, the better therapeutic effects at 1 month after the MSC treatment than that at 1 week are probably due to the enhanced inhibition in the activation of glial cells and loss of neurons.

### Analysis of Differentially Expressed Genes by RNA-Seq Analysis Among MSC_A, MSC_B, and MSC_C Groups After Human Umbilical Cord Mesenchymal Stem Cell Treatment at dpo 3

**FIGURE 7:**
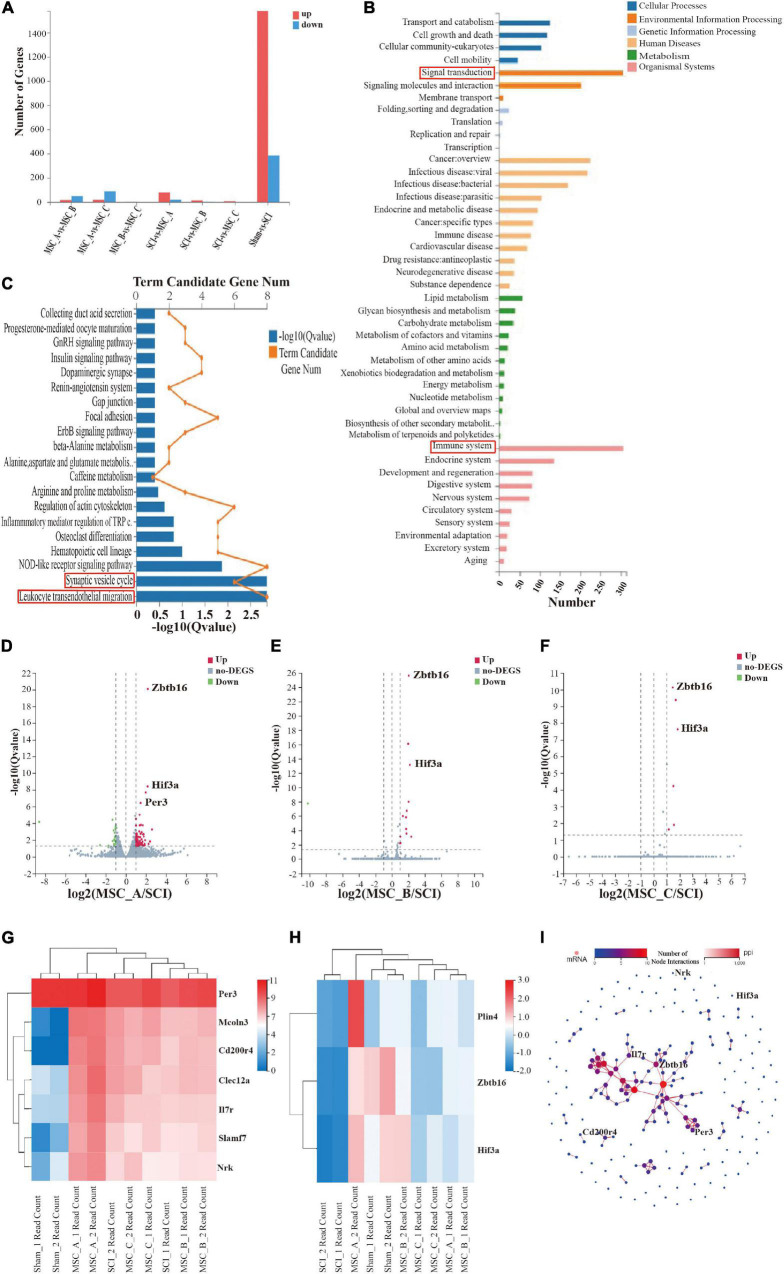
Transcriptome analysis of the spinal cord at 1 week after hUCMSC treatment at dpo 3. **(A)** Statistics of the number of differentially expressed genes. **(B,C)** Kyoto Encyclopedia of Genes and Genomes (KEGG) pathway enrichment of signal pathways. Volcano plot of the MSC_A group **(D)**, the MSC_B group **(E)**, and the MSC_C group **(F)** vs. the SCI group. **(G,H)** The heatmap analysis of the MSC groups vs SCI group. **(I)** PPI showed the protein–protein interaction of the corresponding protein.

The number of differentially expressed genes (DEGs) was analyzed and compared among each group after the transcriptome analysis (Figure 7A). We further performed KEGG pathway enrichment analyses based on the list of DEGs and found that enriched KEGG pathways were mainly related to signal transduction, the immune system, leukocyte transendothelial migration, and the synaptic vesicle cycle (Figures 7B,C). The volcano plot results showed that there were many different genes between the SCI group and Sham group; compared with those in the SCI group, more DEGs existed in the MSC_A group than those in the MSC_B or MSC_C group (Figures 7D–F). A heatmap revealed that the main possible differences in genes involved *Zbtb16*, *Hif3-*α, *Per3*, *Il7r*, *Cd200r4*, and *Nrk* among the MSC groups and the SCI group (Figures 7G,H). The PPI showed the protein–protein interaction of the corresponding protein (Figure 7I).

### Validation of the Expressions of *Zbtb16*, *Hif3-*α, *Per3*, *Il7r*, *Cd200r4*, and *Nrk* in the mRNA Level

**FIGURE 8:**
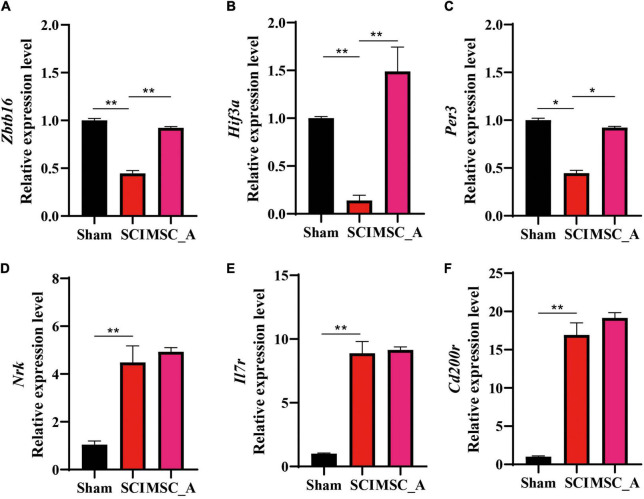
Validation results for *Zbtb16*, *Hif3-*α, *Per3*, *Il7r*, *Cd200r4*, and *Nrk* mRNA by q-PCR. **(A–F)** Validation results for *Zbtb16*, *Hif3-*α, *Per3*, *Il7r*, *Cd200r4*, and *Nrk* mRNA by q-PCR. All data are presented as the mean ± SEM; *n* = 3; *p < 0.05; **p < 0.01.

The above six genes screened by the sequencing analysis were detected using the q-PCR. The results showed that the mRNA expressions of *Zbtb16*, *Hif3-*α, and *Per3* were decreased significantly after SCI. On the contrary, *Il7r*, *Cd200r4*, and *Nrk* mRNA expressions were increased significantly. Compared with those in the SCI group, the increases in *Zbtb16*, *Hif3-*α, and *Per3* mRNA expressions were statistically significant in the MSC_A group (Figures 8A–C), while they were not for *Il7r*, *Cd200r4*, and *Nrk* (Figures 8D–F).

## Discussion

Spinal cord injury is an inestimable central nervous system disease resulting in loss of motor, sensory, and autonomic functions. Given the absence of effective treatment, it remains a challenging medical and social problem at present (O’Shea et al., 2017). According to its pathological mechanism, since there is no specific treatment for primary injury, most of the treatments focus on reducing the impact of secondary injury, including the blood-brain barrier dysfunction, inflammation, neuronal death, oxidative stress, lipid peroxidation, glial scar formation, and so on (Sandrow-Feinberg and Houlé, 2015; Ahuja et al., 2017). Stem cell transplantation has had remarkable effects on repairing SCI in recent years. Among all kinds of stem cells, hUCMSCs showed more potential because of a stronger proliferation ability and lower risk of bacterial/viral infection (Li et al., 2015). MSCs are transplanted into the injured spinal cord through intravenous (i.v.) (Ra et al., 2011; Tanaka et al., 2018), intra-arterial (Watanabe and Yavagal, 2016), *in situ* injection (Lim et al., 2007), or intrathecal routes in past research (Singer et al., 2019). Considering the convenience, safety and ease of operation and repeatability, and the blood-spinal cord barrier damage after SCI, hUCMSCs can colonize in the injured spinal cord after transplantation *via* a vein (Tanaka et al., 2018). In the current study, we chose intravenous injection as the intervention method for hUCMSCs in our experiment. Although whether hUCMSCs can differentiate into neural cells remains controversial, many studies have confirmed that hUCMSCs play a role in inhibiting inflammation after SCI (Yang et al., 2008, 2018; Hu et al., 2010). Considering the individual donors were different, and the preliminary study found different lines of hUCMSCs from 32 donors had different inhibitory abilities on BV2 cell proliferation (Zhang et al., 2021), MSC_A, MSC_B, and MSC_C lines were then selected in this study for animal experiments according to the inhibitory abilities as described above.

In our study, we chose the spinal cord clamp injury model of mice, which was reproducible and simple, to standardize and investigate the effects of intravenous injection of hUCMSCs on the functional recovery after SCI (Plemel et al., 2008; Cheriyan et al., 2014). Since the best time window for cell transplantation remained controversial, we chose to transplant 1 × 10^6^/100 μl cells through the caudal vein at dpo 1, dpo 3, and wpo 2. The BMS results showed that transplantation at dpo 3 worked better than those at the other two time points. More interestingly, the therapeutic effect of the three MSC transplantation groups showed a similar trend as their ability to do inhibition of BV2 cell proliferation when hUCMSC transplantation was performed at dpo 1 and dpo 3. From the 1st to the 6th week, a higher BMS score was shown in MSC_A group than that of the MSC_B or MSC_ C group. There was no obvious difference in therapeutic effects in the three MSC groups when hUCMSC transplantation was performed at wpo 2. It might be that hUCMSCs could not reach the injured spinal cord completely due to the formation of glial scars and repair of the blood-spinal cord barrier started from 2 weeks after SCI (Figley et al., 2014). In addition, the peak of inflammation in the injured spinal cord tissue had passed at 2 weeks after SCI (Beck et al., 2010), when the hUCMSCs might not play a full role in regulating inflammation to repair the SCI.

The results of the Western blotting showed that hUCMSCs had different degrees of ability to reduce the expression of Gfap and Iba1 and increase the expression of NeuN in the protein level compared with those in the SCI group, especially at 1 month after transplantation. The difference in the MSC_A group showed more obvious. The related mRNA expressions were almost consistent with the changing trend of the above proteins. MSC_A intervention lessened the mRNA expressions of *Gfap* and *Iba1* and enhanced the mRNA expression of *NeuN*, which were more significantly than those in the MSC_B or MSC_C group. H&E staining of the spinal cord also showed that hUCMSCs reduced the degree of tissue fragmentation induced by SCI. In the normal spinal cord tissue, most microglia and astrocytes were in a resting state (Rotshenker, 2009), and neurons were distributed in the gray matter of the spinal cord (Fukushima et al., 2011). In our study, many neurons were lost, while large number of astrocytes and microglia were activated after SCI, which might retain at least for 4 weeks. These results were consistent with the previous reports (Li et al., 2020). Moreover, hUCMSC intervention restrained the activation of microglia and astrocytes in the injured area, inhibited the inflammatory response and astrocyte scar formation, and increased the number of neurons in the injured area, which showed more effective at 1 month after transplantation than those at 1 week, especially in the MSC_A group. In short, due to the stronger ability to inhibit the proliferation of BV2 cells *in vitro*, MSC_A intervention not only reduced the loss of neurons, but also inhibited inflammation caused by microglial activation and glial scar caused by astrocyte activation *in vivo*, and finally repaired the SCI better than either the MSC_B or MSC_C group.

To further investigate the underlying mechanisms of the different therapeutic roles, we performed the transcriptome sequencing analysis at 1 week after hUCMSC transplantation and found some major differential genes after the analysis and verification. Zinc finger and BTB domain-containing protein 16 (Zbtb16), known as promyelocytic leukemia zinc finger (Plzf), has been shown to play an important role in neural progenitor cell proliferation and neuronal differentiation during development (Sobieszczuk et al., 2010). A study reported that neurons in the neocortex were reduced in *Zbtb16* KO mice at neonatal stages, while the numbers of dendritic spines and microglia increased, and oligodendrocyte developmental abnormalities were found in *Zbtb16* KO adult mice (Usui et al., 2021). Moreover, Zbtb16 repressed the Toll-like receptor 4-dependent inflammatory signaling pathway and nuclear factor and had the function of restraining autophagy and apoptosis (Ibrahim et al., 2021; Wei et al., 2021). In our study, the mRNA expression of *zbtb16* was decreased significantly after SCI, while reversed after injection of MSC_A, indicating that MSC_A improved the microenvironment after SCI, probably by promoting the formation of neurons and inhibiting the inflammatory response, which needs further investigation.

Hypoxia-inducible factors (HIF-α and HIF-β) play a key role in adaptive mechanisms to respond to hypoxic stress. HIF-1α, HIF-2α, and HIF-3α are three paralogs of HIF-α, which is a master regulator controlled by oxygen availability (Cai et al., 2020; Qu et al., 2021). Data have suggested that HIF-1α and HIF-2α are activated in hypoxia and involved in the inflammatory response (Guo and Chen, 2020). All specific functions of the *HIF3*α gene are still unclear. A study has shown that some *HIF3*α isoforms could suppress *HIF1*α and *HIF2*α expressions (Drevytska et al., 2018). The *HIF3*α gene is involved not only in inflammation but also in hemangiogenesis associated with the injury resolution and tissue repair. Francesca Cuomo et al. (2018) found *Hif3*α was expressed in the inflamed tissue and might contribute to the repair process by participating in the process of angiogenesis. Its acceleration in the presence of allogeneic MSCs was endowed with immunomodulatory and secretory properties (Cuomo et al., 2018). In the present study, SCI lessened the mRNA expression of *HIF3*α, which could be reversed by transplantation of MSC_A. These results reveal that the repair of the injured spinal cord by MSC_A injection is likely to be related to the promotion of hemangiogenesis through *HIF3*α.

The *Per3* gene is mostly tied to the circadian rhythm, which influences neuropsychiatric diseases and pathological processes of tumors, such as colorectal cancer and prostatic cancer (Zhang et al., 2017; Noda et al., 2019). It has been shown that *Per3* can inhibit the signal pathway of Notch to regulate drug resistance of tumors (Cai et al., 2018). Previous studies reported that Notch signaling participated in microglia-mediated inflammatory responses (Quillard and Charreau, 2013) and restraining the Notch pathway could alleviate SCI-induced microglia inflammation and neuronal apoptosis (Jian et al., 2020; Zhou et al., 2020). In the present study, SCI reduced the mRNA expression of *Per3*, which could be reversed by MSC_A intervention. These results suggest that the effect of MSC_A on SCI repair might be related to the inhibition of the inflammatory reaction and the reduction of neuronal apoptosis.

Cytokine Il-7 and its receptor, Il-7r, are critical for innate and adaptive immune responses, which are related to differentiation and survival of T cells (Passtoors et al., 2015). It has been showed that Il-7–Il-7r signaling was associated with chronic inflammatory diseases and participated in the inflammatory response in high levels (Barata et al., 2019; Belarif et al., 2019). Previous research proved that the IL-7 expression increased after SCI, and reducing IL-7 could improve the prognosis of SCI (Bao C. et al., 2018; Bao C. S. et al., 2018). In the central nervous system, Cd200 is primarily distributed in neurons (Webb and Barclay, 1984), while Cd200r4, as one subtype of Cd200r, is expressed in microglia (Hoek et al., 2000). The Cd200-Cd200r interaction plays a pivotal role in inhibiting the activation of microglia and releases of the proinflammatory mediators (Zhao et al., 2019). Neurons are found to have a self-protection mechanism in principle by regulating microglia function through their Cd200 expression binding to Cd200r on the microglial surface (Zhao et al., 2019). Previous study proved CD200 could modulate the SCI neuroinflammation and outcome through CD200R1 (Lago et al., 2018). The increase in *Cd200r4* after SCI may be related to inflammatory response and the self-protection mechanism of neurons. Nrk (Nik-related kinase), an X-linked protein kinase in the germinal center kinase family, is required for placental development due to its role as a crucial modulator of cell proliferation (Lu et al., 2020). Studies have shown that Nrk induced the activation of the c-Jun N-terminal kinase (JNK) pathway through the MEKK1 and MKK4 kinase cascade and finally promoted apoptosis (Nakano et al., 2000; Kakinuma et al., 2005). The high expression of *Nrk* may be involved in apoptosis after SCI. In the current study, we found the increase of *Il-7r*, Cd200r4, and *Nrk* expressions after SCI, while there was no statistical difference between SCI group and MSC_A group, indicating that these three genes are probably involved in the pathological process of SCI.

Based on our results, hUCMSCs transplantation for acute SCI promotes the recovery of motor function. MSC_A had the best therapeutic effect among three cell lines, which was closely related to its inhibitory role in microglia. Furthermore, the DEG genes including *Zbtb16, Per3*, and *Hif3a* were found to be involved in the SCI and might play roles in MSC transplantation. These results suggest that the effect of hUCMSCs transplantation on acute SCI depends on their inhibitory abilities to inflammation reaction after nerve injury, which may help to shape future use of hUCMSCs combined with improving the effectiveness of clinical transformation.

## Data Availability Statement

The original contributions presented in the study are included in the article/supplementary material, further inquiries can be directed to the corresponding authors.

## Ethics Statement

The studies involving human participants were reviewed and approved by the Ethics Committee of Shanghai Tongji Hospital. The patients/participants provided their written informed consent to participate in this study. The animal study was reviewed and approved by the Ethics Committee of Shanghai Tongji Hospital.

## Author Contributions

YS, ZWu, BM, and LC conceived and designed the study. ZWa, XZ, ZWu, and YL performed the experiments and analyzed the data. All authors contributed to the discussion. XZ and BM wrote the manuscript. All authors contributed to the article and approved the submitted version.

## Conflict of Interest

The authors declare that the research was conducted in the absence of any commercial or financial relationships that could be construed as a potential conflict of interest.

## Publisher’s Note

All claims expressed in this article are solely those of the authors and do not necessarily represent those of their affiliated organizations, or those of the publisher, the editors and the reviewers. Any product that may be evaluated in this article, or claim that may be made by its manufacturer, is not guaranteed or endorsed by the publisher.
